# Decoding basic emotional states through integration of an fNIRS-based brain–computer interface with supervised learning algorithms

**DOI:** 10.1371/journal.pone.0325850

**Published:** 2025-07-14

**Authors:** Ayşenur Eser, Sinem Burcu Erdoğan

**Affiliations:** 1 Faculty of Engineering and Natural Sciences, Department of Biomedical Engineering, Acıbadem Mehmet Ali Aydınlar University, İstanbul, Türkiye; Air University, PAKISTAN

## Abstract

Automated detection of emotional states through brain-computer interfaces (BCIs) offers significant potential for enhancing user experiences and personalizing services across domains such as mental health, adaptive learning and interactive entertainment. Within this advancing field, the aim of this study was to test the feasibility of a functional near-infrared spectroscopy (fNIRS)-based BCI system for accurate prediction and objective identification of three fundamental emotional states that involved positive, negative and neutral conditions. Consequently, the efficacy of fNIRS signals in predicting the valence of standardized stimuli from the International Affective Picture System (IAPS) was assessed. fNIRS data were collected from twenty healthy participants while images from the IAPS database were presented. The images varied in both valence (i.e., positive, neutral, negative) and arousal (i.e., high, low) level. Hemodynamic responses of prefrontal cortical (PFC) regions were recorded with a twenty-two channel system. Twenty fNIRS derived time domain features were extracted from HbO time traces of each channel corresponding to each stimulus period. Classification performances of three machine learning algorithms, namely the k-Nearest Neighbors (kNN), Ensemble (Subspace kNN) and Support Vector Machines (SVM), in two class and three class classification of positive, neutral and negative states were evaluated with ten runs of a tenfold cross-validation procedure through splitting the data into test, train and validation groups at each run. Three class classification performances of all algorithms were above 90% in terms of accuracy, sensitivity, specificity, F-1 score and precision metrics while two class accuracy performances of all algorithms were above 93% in terms of each performance metric. The high-performance classification results highlight the potential of fNIRS-based BCI systems for real-time, objective detection of basic emotional states for daily life and clinical applications. fNIRSbased BCIs may show promise for future developments in personalized user experiences and clinical applications due to their practicality and low computational complexity.

## Introduction

Emotion recognition may play a crucial role in a variety of clinical and daily applications such as mental health assessment, evaluation of phenomenal states during human–computer interactions and adaptive technologies [1,2]. Accurately detecting and interpreting human emotions enhances user experiences and facilitates personalized responses in domains such as education, entertainment, and clinical diagnostics [3,4]. Conventional emotion recognition methods often rely on peripheral physiological signals such as voice modulation, facial expressions and body language. However, these signals can be intentionally manipulated or affected by external factors, resulting in reduced reliability [5,6].

Functional near-infrared spectroscopy (fNIRS) has emerged as a promising neuroimaging modality for emotion recognition due to its non-invasive nature, portability, and cost-effectiveness [7–9]. fNIRS measures hemodynamic responses associated with neuronal activation by detecting changes in oxygenated (HbO) and deoxygenated hemoglobin (HbR) concentrations [10]. This capability allows for real-time monitoring of neurophysiology in naturalistic settings, making it suitable for developing wearable brain–computer interfaces (BCIs) aimed at emotion detection [11,12].

The prefrontal cortex (PFC) plays a crucial role in neural processing, regulation and interpretation of emotional stimuli [13–15]. Previous studies have demonstrated that emotional stimuli elicit distinct hemodynamic responses in the PFC which can be measured with fNIRS. For instance, Balconi et al. (2009) reported increased HbO levels in the PFC when emotional stimuli from the International Affective Picture System (IAPS) were displayed to the participants [16]. Similarly, Hu et al. (2019) provided fNIRS evidence for differentiating positive emotions based on hemodynamic responses [17].

Machine learning (ML) algorithms have been widely adapted to fNIRS data for emotion recognition tasks, by use of techniques such as Random Forest, Support Vector Machines (SVM) and k-Nearest Neighbors (kNN) classifiers [18–24]. These methods leverage features extracted from fNIRS signals, such as mean, variance, skewness, and kurtosis, to distinguish between different emotional conditions [25]. Al-Shargie et al. demonstrated the effectiveness of SVM classifiers in distinguishing high level stress from low level stress using fNIRS signals from the PFC [26]. Similarly, Hu et al. employed kNN and SVM algorithms to classify positive and negative emotions, achieving high accuracy rates [17]. Trambaiolli et al. (2021) also demonstrated subject-independent binary classifications among positive, negative, and neutral emotional states using time domain fNIRS features and LDA achieving accuracies in the range of 65–71% [27]. Lastly, Ruotsalo et al. (2024) classified emotional states based on valence and arousal quadrants using fNIRS-derived hemodynamic responses, though achieving relatively modest accuracies of approximately 48% for four-category classification at the individual level [28]. These studies suggest the feasibility of integrating traditional machine learning approaches with fNIRS derived data given their relatively low computational requirements and ability to perform well with small datasets, which is essential for real-time BCI applications [10,29]. However, challenges such as individual differences in hemodynamic responses and the need for obtaining robust and high classification performances with standardized feature selection strategies persist, affecting the generalizability of the models across diverse populations [30,31].

Recent studies have also explored the utility of deep learning techniques for emotion recognition using fNIRS data [32,33]. For instance, Si et al. (2024) tested the classification performance of a transformer-based deep learning model for distinguishing three valence states (i.e., positive, neutral, negative) from fNIRS and EEG signals, achieving approximately 84% accuracy in a cross-subject scenario [34]. Another study by Si et al. (2023) similarly classified positive, neutral, and negative emotions by combining convolutional neural networks (CNNs) and mean oxygenation time-domain features, reporting a cross-subject accuracy of around 75% [11]. However, it should be noted that deep learning models often require large datasets and substantial computational resources, which can be challenging in practical applications [10,35]. Moreover, the complexity and cost associated with these models can limit their scalability and usability in real-world settings [33].

Within this advancing field of integrating BCIs with neurophysiological data, the aim of this study is to evaluate the feasibility of using an fNIRS-based BCI system for accurate prediction and objective identification of three fundamental emotional states that involve positive, negative and neutral conditions. For this purpose, we tested the efficacy of training three distinct machine learning models with fNIRS-derived time domain features and assessed their two-class and three-class classification performances. Our work extends the current line of research by focusing exclusively on neuronally induced hemodynamic signals obtained noninvasively in naturalistic settings without relying on i) behavioral or clinical features of potentially biased origin or ii) impractical, hybrid methods that necessitate multi-modal data integration. When compared to previous studies, the proposed machine learning based methodology demonstrates robust classification performance (exceeding 90% accuracy) in identification of positive, neutral, and negative emotional states by use of simple time domain features obtained from a single, practical, and ergonomic sensor modality. This approach highlights the feasibility and effectiveness of combining fNIRS-only features with ML for accurate emotional state detection, resulting in reduced computational complexity and cost while showing promise for real time applications.

## Materials and methods

### Participants

Twenty healthy subjects (gender distribution: 10 males, 10 females, mean age = 23, standard deviation = 1.31, age range: 20–26 years) participated in the study. All participants were right-handed, had normal or corrected-to-normal vision, and had no history of neurological or psychiatric disorders. Three subjects were excluded from the study due to poor optical signal quality, resulting in a final cohort size of 17 subjects (10 males, 7 females). The study protocol was approved by the Ethics Committee of İstanbul Medipol University, İstanbul, Türkiye. All subjects gave verbal consent to the experimenter prior to the experiment. Experiments were conducted between 01/11/2022 and 01/03/2023. The experiments were carried out in accordance with the Declaration of Helsinki.

### Experimental protocol

The experimental protocol involved passive viewing of emotional pictures on a computer screen in a dimly-lit, quiet room while fNIRS recordings were taken with a 22 channel fNIRS system from the PFC of each participant. The participants were informed about the experimental protocol at the onset of each experimental session and they were requested to sit relaxed and avoid head movements during the experiment. The emotional pictures used in this study were selected from the International Affective Picture System (IAPS) database, which provides standardized pictures with predefined valence and arousal scores [36]. Sixty stimuli were chosen from the IAPS database, consisting of 30 pleasant and 30 unpleasant pictures. The pleasant pictures had a mean valence score of 7.37 ± 1.61 and arousal score of 6.40 ± 2.2 while the unpleasant pictures had a mean valence score of 2.32 ± 1.56 and a mean arousal score of 6.42 ± 2.2. Selected IAPS stimuli pictures were: (a) pleasant: 8200, 5260, 8206, 8080, 8470, 8490, 8492, 8370, 8499, 8501, 8380, 8260, 5833, 8400, 5470, 8030, 8161, 8034, 8163, 8170, 8300, 7405, 2034, 8180, 5621, 8185, 8186, 4220, 5629, 8190; (b) unpleasant: 6021, 3212, 3213, 6540, 6415, 9620, 9630, 9250, 9635.1, 9252, 8485, 3110, 6570.1, 6834, 2683, 3005.1, 9921, 6212, 9412, 9413, 3400, 9163, 9810, 9300, 9050, 6370, 9321, 6250.1, 9075, 3195.

**Fig 1:**
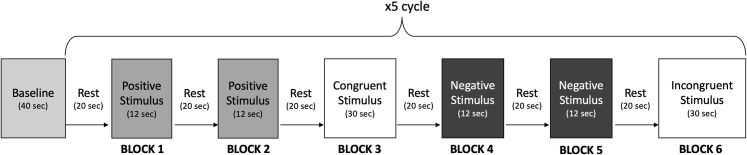
Experimental Protocol. Each experimental session consisted of presentation of positive and negative valence emotional stimuli blocks besides congruent and incongruent Stroop trial blocks. The depicted stimuli presentation cycle was repeated 5 times. Stroop trial blocks were placed in between different consecutive emotional stimuli blocks in order to wash out cognitive and emotional effects of previously presented emotional stimuli block.

Each experimental session began with a 40-second baseline recording, followed by two consecutive blocks consisting of positive valence emotional stimuli trials, a congruent Stroop trial block, two consecutive blocks consisting of negative valence emotional stimuli trials, and an incongruent Stroop trial block. This sequence of stimuli presentation was repeated 5 times to ensure robust data collection (Fig 1). Stimuli blocks which involved presentation of pleasant pictures were labelled as ‘positive’ while stimuli blocks which involved presentation of unpleasant pictures were labelled as ‘negative’. Hence, the experimental protocol involved 10 blocks of positive valence stimuli and 10 blocks of negative valence stimuli. Each emotional stimuli block lasted for 12 seconds where three consecutive images with similar valence and arousal scores were displayed for 4 seconds each. Each Stroop block lasted for 30 seconds, consisting of six congruent or incongruent trials presented for 5 seconds each. Within the congruent Stroop trial blocks, each trial consisted of presentation of a color name where the ink color of the word matched its meaning, while for the trials presented within the incongruent Stroop blocks, the ink color and word meaning did not match. All stimulus blocks were separated by 20-second rest intervals during which subjects were asked to focus on the asterisk symbol presented on the screen.

Stroop task blocks were presented in between consecutive emotional stimuli blocks with different valences in order to i) eliminate the potential after-image residual emotional effects of previously presented emotional stimuli block, ii) engage participants cognitively, and iii) assess their attentional processes and interference effects. More specifically, Stroop trial blocks were introduced in between consecutive emotional stimuli blocks with different valences to enable a washout effect of the potential after-image residual emotional effects of previously induced affective state and to ensure that participants enter each new emotional condition from a relatively neutral baseline. Engaging participants in a cognitively demanding task such as the Stroop (i.e., color–word matching) was considered to redirect the attentional resources away from the previously encountered emotional stimuli, thereby “washing out” residual emotional processing. This approach would improve the discriminability of subsequent emotional responses by reducing confounding influences from previously presented emotional stimuli of different valences. Moreover, the Stroop task was considered to enhance attentional engagement and cognitive control through task-switching, which would assist the participants in remaining consistently focused throughout the session—minimizing potential confounds such as habituation. By including Stroop tasks in this manner, we follow a strategy used in other psychophysiological studies where short, cognitively neutral interventions effectively reset the participant’s internal state before the next experimental condition. This approach is consistent with the findings of Visalli et al. (2023), who showed that the proactive- and reactive-control demands of an intervening emotional-priming Stroop task effectively abolish residual affective interference [37].’

The selected unpleasant and pleasant pictures were labelled as ‘positive’ or ‘negative’ valence stimuli based on their standardized IAPS valence scores. Subjective ratings of each presented picture were also collected after the experiments by using a Likert Scale to check whether i) the subjects produced arousal behavior in accordance with IAPS database scores and ii) their emotional responses could be represented by IAPS database. IAPS subjective ratings of arousal for each picture were obtained with the Likert scale, using a 9-point version [36]. This scale ranged from 1 to 9, representing states between low and high arousal conditions. Indeed, a good correspondence between IAPS arousal scores and the subjective arousal rating scores were found.

### fNIRS data acquisition and preprocessing steps

**Fig 2:**
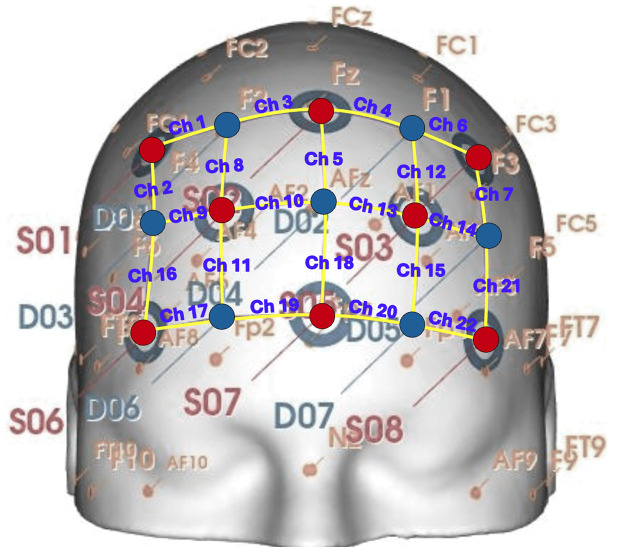
Probe configuration and montage design. Each red dot corresponds to a light source while each blue dot represents a detector. Channels are formed between adjacent source-detector pairs, represented with yellow lines and labelled accordingly. Location of all channels are projected onto their EEG 10-20 coordinates.

fNIRS data were collected using a NIRSport system (NIRx Medical Technologies, LLC, Berlin, Germany), with 8 light sources and 7 detectors strategically positioned over the prefrontal cortex (PFC) forming 22 channels. The light sources emitted continuous wave near-infrared light at wavelengths of 760 nm and 850 nm. Channel forming optodes were placed 3 cm apart to ensure optimal signal penetration and coverage of the target brain regions was ensured with 22 channels. Sampling rate of the system was 7.81 Hz, and data were recorded continuously throughout the experiment. A schematic diagram of the probe design is presented in Fig 2 where EEG 10–20 coordinates corresponding to each optode is provided.

Preprocessing of fNIRS raw data was carried out by use of customized MATLAB scripts (Mathworks, Natick, MA, USA) and Homer3 toolbox [38]. The light intensity signals of each channel were converted to optical density (OD) values using the hmrR_Intensity2OD function. The OD data underwent motion correction using the hmrR_MotionCorrectSplineSG function with a parameter setting of p = 0.99 and a frame size of 10 seconds. Following motion correction, the OD data were converted to concentrations of oxygenated hemoglobin (HbO) and deoxygenated hemoglobin (HbR) using the hmrR_OD2Conc function where the differential pathlength factor (DPF) was selected as 1 for each wavelength. Bandpass filtering was applied with high and low cutoff frequencies of 0.01 Hz and 0.5 Hz to remove low-frequency drifts and high-frequency noise induced by heartbeat. Additionally, correlation-based signal improvement (CBSI) was applied to the bandpass-filtered data to further reduce motion artifacts and improve signal quality. Upon completion of preprocessing steps, HbO time series data of each channel were truncated into negative and positive stimuli blocks with a pre-stimulus baseline of 3 seconds and a post-stimulus duration of 10 seconds resulting in 25 second time traces for each stimulus condition. Neutral state data were obtained by truncating the rest interval HbO time series data of each channel into 20 second segments. Channel specific HbO signals corresponding to each stimulus condition were detrended prior to further analysis. Each emotional condition (i.e., negative, positive, neutral) had a total size of 3740 samples (22 channels*17 subjects *10 trials).

### Feature extraction and classification

Twenty hemodynamic time domain features were extracted from the HbO time traces of each channel and stimulus block. The extracted features were 1) maximum, 2) range (i.e., amplitude difference between maximum and minimum signal values), 3) signal to noise ratio, 4) peak time, 5) skewness, 6) kurtosis, 7) variance, 8) median, 9) root mean square, 10) area under the curve and step response characteristics obtained with ‘stepinfo’ function implemented in MATLAB 2017B (The Mathworks Inc., Natick, MA, USA) which included 11) rise time, 12) settling time, 13) settling min, 14) settling max and 15–20) coefficients of a fifth-degree polynomial which was fitted to each channel and stimulus specific truncated time series HbO data.

**Fig 3:**
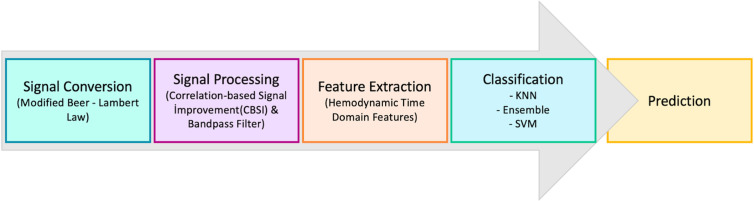
Overview of the Data Analysis Pipeline. The pipeline consists of four main stages: (1) Conversion of raw optical signals into relative changes in HbO concentration using Modified Beer-Lambert Law; (2) Preprocessing of HbO signals through Correlation-Based Signal Improvement (CBSI) and a Butterworth bandpass filter; (3) Feature Extraction, where hemodynamic time-domain features are extracted(e. g. mean, variance, kurtosis…); and (4) Classification through machine learning algorithms which include k-Nearest Neighbors (KNN), Ensemble, and Support Vector Machine (SVM).

The extracted features were utilized to train three supervised machine learning algorithms: Support Vector Machine (SVM), Ensemble (Subspace KNN), and k-Nearest Neighbors (kNN). These algorithms were selected based on their ability to handle small sample sizes effectively, their demonstrated accuracy in previous studies involving similar fNIRS features, and their low computational cost. Additionally, they have shown robust performance in classifying emotion-related data in neuroimaging research. Overall, as shown in Fig 3, the data analysis pipeline consists of several key stages, including conversion of optical signals into HbO data, preprocessing of HbO signals, feature extraction, and classification, which ultimately allowed prediction of each emotional state (i.e., positive, negative and neutral).

For all three algorithms, labelled data underwent a random partitioning process where 80% of the dataset was preserved for training, 10% for validation, and 10% for testing. Classification performances of each algorithm were evaluated with 10 runs of a tenfold cross-validation procedure for two class and three class classification of positive, neutral and negative states.

All classification models were developed using MATLAB’s Classification Learner toolbox. Each classification method was optimized through a grid search process. The Ensemble method (Subspace kNN) combined multiple kNN models trained on different subsets of features. This approach resulted in a more reliable and generalizable model compared to using a single kNN classifier [39].

Briefly, the Ensemble (Subspace kNN) method combined decisions obtained from multiple kNN models, where each base kNN model was trained on a randomly selected subset of the original feature space. The subspace feature set utilized for training each base kNN classifier involved 10 randomly selected features. 30 base kNN learners were trained with 10 randomly selected features and the individual predictions of each kNN classifier were combined to form a final classification outcome through a majority voting approach. Selecting a random feature subset for each of the 30 base kNN learners allowed for some overlap across the feature subsets while ensuring sufficient diversity. Training multiple base kNN learners on smaller feature subspaces and obtaining a final decision for the Ensemble through a majority voting approach mitigated the risk of overfitting across the learners while reducing each classifier’s exposure to noise or less relevant features [39]. For each base kNN learner model, the initial data set consisted of 11,220 labelled samples (22 channels * 10 trials * 17 participants * 3 classes) before splitting into training, validation and testing sets. Specifically, each of the three emotional conditions (i.e., positive, negative, neutral) contributed 3,740 samples, derived from 17 participants * 22 channels * 10 trials. These condition-specific samples are concatenated, yielding a total of 11,220 rows with 11 columns (10 features + 1 label column) in the final feature matrix. For each base kNN learner of the Ensemble (Subspace kNN) model, the input feature matrix underwent an 80:10:10 random split for training, testing and validation purposes. Consequently, although each base kNN learner in the Ensemble spanned only a randomly selected subspace of features and each learner was trained on 8,976 samples (i.e., 80% of the samples are reserved for training) through the cross-validation procedure, ensuring a consistent and comprehensive use of the available data.

For the SVM algorithm, a 3^rd^ degree polynomial kernel was chosen. The hyperparameters were configured with a box constraint of 1 and automatic kernel scaling option (i.e., MATLAB automatically chooses the optimal bandwidth for the kernel while balancing margin maximization and misclassification tolerance). A “One‐vs‐One” multiclass scheme was implemented so that each of the three emotion classes would be distinguished by pair-wise binary SVMs. The choice of a cubic kernel (rather than a linear or quadratic one) was motivated by the need to capture complex, nonlinear relationships among the 20 fNIRS time‑domain features while avoiding overly flexible, higher‐order polynomial surfaces, which may result in overfitting. The cubic kernel’s flexibility was considered to be useful for distinguishing closely related states such as neutral vs. positive—while the box constraint and cross‑validation controlled for overfitting.

A single kNN model was trained by using all the features in the dataset and a relatively small number of neighbors (k = 1). The choice of a low number of neighbors allows kNN algorithm to be more sensitive to local variations in feature space. By focusing on only the nearest neighbor, the kNN method could capture the subtle distinctions among samples at its best.

Accuracy, precision, sensitivity, specificity, and F1-score of each algorithm were evaluated as the performance metrics by comparing the actual and predicted labels of validation data [25]. For a statistical comparison of the classification performances of all algorithms, the mean and standard deviation of each performance metric were computed over multiple runs for each algorithm (Table 1). For each performance metric, a one-way repeated measure ANOVA (main effect: classifier type) was initially performed to evaluate whether any significant difference existed among the performances of the three classification algorithms. Post hoc analysis was performed using paired t-tests, with the Bonferroni corrected significance threshold set at p < 0.05 for the following contrasts: SVM > kNN, SVM>Ensemble, Ensemble>kNN. Each performance metric (e.g., accuracy, specificity, recall, precision, and F1-score) was evaluated across the algorithms to determine whether any algorithm demonstrated statistically significant superiority in performance compared to the others.

**Table 1:** Classification performances of Ensemble, SVM, and kNN algorithms. For each performance metric, the mean value across all runs and standard deviation of the mean value is reported in percentages (%).

Method	Accuracy Performance (%, Mean± STD)			
	3 – Class	2 – Class		
	Neg/ Pos/ Neutral	Neg vs Pos	Neg vs Neutral	Pos vs Neutral
**Ensemble**	93.11 ± 0.23	95.54 ± 0.16	95.85 ± 0.37	94.99 ± 0.23
**SVM**	94.32 ± 0.15	96.54 ± 0.18	96.64 ± 0.39	95.56 ± 0.10
**KNN**	90.33 ± 0.24	93.56 ± 0.16	93.73 ± 0.22	93.42 ± 0.16

## Results and discussion

In the presented work, an fNIRS-based, computationally efficient machine learning approach is proposed for classifying three distinct emotional states (positive, negative, and neutral) by use of time-domain hemodynamic features obtained from the prefrontal cortical regions. To accomplish this goal, we compared the classification efficacies of kNN, Ensemble (Subspace kNN), and SVM algorithms, with the ultimate purpose of identifying a model that offers an optimal balance among robustness, accuracy, and generalizability under real-world conditions. Table 1 presents accuracy performances of Ensemble, SVM, and kNN algorithms for 2-class and 3-class classification of neutral, negative and positive states. All three methods achieved 2 class accuracy performances above 93%, with Ensemble and SVM methods exceeding 95%. The SVM classifier yielded the highest performance, with accuracies surpassing 90% for three-class classifications and 93% for two-class classifications. The statistical analysis confirmed that SVM significantly outperformed the other methods, with p-values < 0.05 after Bonferroni correction.

**Fig 4:**
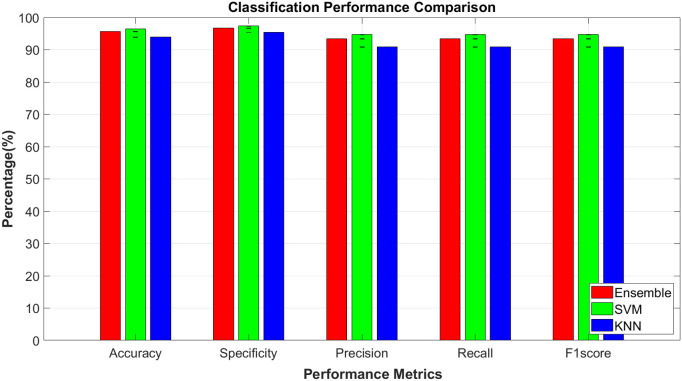
Classification performances of Ensemble (Subspace KNN), SVM, and KNN algorithms when trained with time domain HbO features.

All algorithms demonstrated strong performance, with accuracy, precision, specificity, recall, and F1-score exceeding 90%, and specificity scores above 93%. Notably, the SVM classifier achieved the highest scores across all metrics (Fig 4), with an accuracy of 94.1% ± 1.2% (p < 0.00125), precision of 92.7% ± 1.1% (p < 0.00125), recall of 93.5% ± 1.0% (p < 0.00125), specificity of 96.3% ± 0.9% (p < 0.00125), and an F1-score of 93.8% ± 1.0% (p < 0.00125). Paired two tailed t-test results demonstrated that the performance of SVM was statistically significantly higher than the performance of the Ensemble (Subspace kNN) method in terms of accuracy, precision, specificity, recall, and F1-scores (p < 0.00125). Similarly, SVM had statistically significantly higher classification performance than kNN in terms of accuracy, precision, specificity, recall, and F1-scores (p < 0.00125). A performance comparison of the Ensemble and kNN classifiers indicated that the Ensemble Subspace kNN methodology had a statistically significantly higher classification performance than the kNN method in terms of accuracy, precision, specificity, recall, and F1-scores (p < 0.00125). The high specificity observed across all classifiers indicates a low rate of false positives, suggesting that the features extracted from the HbO signals were highly discriminative of the different emotional states. The results suggested that time-domain features, such as mean, variance, skewness, and kurtosis were able to capture essential characteristics of the hemodynamic responses associated with emotional processing [1,17,40]. These features likely contributed to the high precision and recall rates, as they effectively differentiated between the physiological patterns of each emotional state.

**Fig 5:**
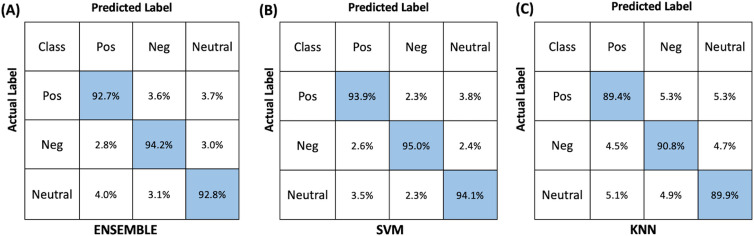
Confusion matrices illustrate the true positives (highlighted in blue) and false negatives for (A) Ensemble, (B) SVM, and (C) kNN when trained, tested, and validated with population level time domain fNIRS features.

Fig 5 illustrates the confusion matrices for each classification algorithm, highlighting the true positive and false negative prediction percentages of each algorithm. The highest true-positive prediction rates were observed for negative stimuli, followed by neutral and positive stimuli across all algorithms. This finding aligns with previous research which reported that negative emotional stimuli often elicit stronger and more distinct hemodynamic responses in the PFC compared to positive stimuli [16,17,41]. Negative emotions are associated with increased activation in specific brain regions, leading to more pronounced HbO changes that are easier for classifiers to detect [13].

The consistently low false attribution rates (less than 11% for all algorithms and emotional states) suggest that the selected fNIRS features were both distinctive and physiologically informative. This result is significant because it demonstrates the effectiveness of the feature extraction process in capturing the underlying neural correlates of emotion, which is critical for reliable emotion recognition systems [1,11,33]. The fact that the utilized time domain features demonstrated a high classification performance with all three classifiers regardless of their different mathematical architectures, also emphasizes the potential and practicality of utilizing machine learning-based methods with fNIRS-derived neuronal features to classify emotional states linked to changes in frontal lobe function [42,43].

The probability of falsely classifying neutral states as positive (SVM: 3.5%, kNN: 5.1%, Ensemble: 4.0%) or vice versa was slightly higher than the false attribution of negative states to positive states. This trend may be due to the overlapping neural activations and subtle differences in hemodynamic responses between neutral and positive emotions. Positive and neutral stimuli can sometimes evoke similar patterns of brain activity, especially if the positive stimuli are not highly arousing [12,17,44,45]. This overlap can make it more challenging for classifiers to distinguish between these two states compared to the more distinct negative state.

In many conventional fNIRS studies, the effect of systemic physiological fluctuations (particularly those associated with respiration, heart rate, and blood pressure variability) on the recorded hemodynamic signals are reduced by applying a band pass filter with lower high cut-off frequency (i.e., 0.2 Hz) or by regressing out auxiliary measured signals such as scalp hemodynamics, heart rate and/or respiratory signals. However, we took into account the possibility that the systemic physiological effects associated with different emotional states may contain distinctive information regarding stimulus-induced hemodynamic changes. Indeed stimulus-specific hemodynamic signatures for differentiating different emotional states may stem not only from neuronal but also from systemic physiological effects, including slow hemodynamic signals that do not strictly follow canonical respiration or heart-rate patterns. To examine whether including the 0.3 Hz respiratory band would degrade or potentially enhance our ability to classify the hemodynamic processing of different stimuli, we systematically evaluated the efficacy of obtaining features after multiple preprocessing pipelines. These preprocessing pipelines varied in their combination of i) motion artifact removal methods (e.g., spline interpolation, correlation-based signal improvement), and ii) bandpass cut-off frequencies (e.g., 0.01–0.2 Hz, 0.01–0.3 Hz, 0.01–0.5 Hz). After training and testing the performance of each feature extraction pipeline with our supervised learning algorithms, we found that retaining the 0.5 Hz upper cut-off yielded the highest classification accuracy across multiple runs of cross-validation procedure for all algorithms. While a 0.3 Hz respiratory component could introduce bias for analyses that target precise hemodynamic activity localization (e.g., in the form of false positive and false negative activity localization) or connectivity mapping purposes (e.g., in the form of spurious correlations for functional connectivity analyses), our comparative results suggest that excluding this frequency component might discard subtle yet valuable stimulus-related hemodynamic signatures. Selecting the 0.01–0.5 Hz range in our final preprocessing pipeline struck a favorable balance: it attenuated low frequency neuronally induced hemodynamic effects while preserving potential respiratory-frequency contributions that, in practice, did not degrade classification performance and may have captured meaningful neural or systemic covariations relevant to the emotional stimuli.

### Performance comparison

Our results indicated that SVM performed statistically significantly better than the Ensemble (Subspace kNN) and the kNN algorithms in terms of accurate classification of the three emotional states tested in the present study. The superior performance of SVM in both two-class and three-class classification scenarios suggests that SVM is particularly well-suited for fNIRS-based emotion recognition tasks. This result can be related to several factors associated with the nature of fNIRS data and the characteristics of the SVM algorithm.

fNIRS datasets display high-dimensional, nonlinear characteristics besides involving inherent complex hemodynamic patterns that are associated with the neural processing of emotional stimuli [7,17,46,47]. SVMs are particularly effective in handling high-dimensional, complex datasets, such as fNIRS signals, primarily due to their ability to efficiently manage dimensionality and nonlinearity issues through their margin-based learning principle and the kernel trick property. Specifically, the margin-based principle seeks the optimal hyperplane that maximizes the geometric margin (i.e., distance) between classes [48]. By relying only on the support vectors that are formed between data points closest to the decision boundary, the margin-based principle makes SVM naturally robust to noisy data points, as the majority of the data do not influence positioning of the decision boundary. Hence, outliers or noisy points have no effect on the decision boundary once the margin is established. In contrast, kNN based classification approaches are distance-based methods which weigh decisions from all samples equally. This property causes decisions obtained from kNN based approaches to be more affected by noisy data points or sample size imbalances as they treat all data points equally.

fNIRS signals exhibited non-linear relationships between features and emotional states due to the intricate hemodynamic responses in the brain [29,49]. With kernels, SVMs can model very complex, nonlinear decision boundaries, even in very high dimensions. In our study, we used a kernel-based SVM which allowed for mapping input features into higher-dimensional spaces where non-linear patterns became linearly separable [50]. The kernel trick feature of SVM is especially advantageous for fNIRS data, where complex, overlapping patterns of HbO features can arise from different cortical regions and physiological states [17,29]. Consequently, even subtle distinctions among emotional responses may become easier to identify. Meanwhile, KNN based approaches have no inherent mechanism for learning nonlinearity as their decisions rely solely on local distance between neighbor data points.

From a dataset size perspective, SVM tends to handle relatively small sample sizes robustly, partly due to its focus on maximizing margins [48,51]. Margin maximization prevents overfitting by ensuring that only the critical points closest to the boundary define the classification model through their geometric arrangement, making it resilient even in sparse, high-dimensional feature spaces. This feature makes SVM highly resistant to overfitting—particularly valuable in neuroimaging datasets such as fNIRS, where sample sizes are limited, and data complexity is high. Ensemble Subspace kNN can likewise mitigate overfitting by aggregating base learners, yet if certain random feature subsets are less discriminative, those base learners may yield weaker local decision boundaries. These marginal drops in performance across multiple base learners may have diminished the Ensemble’s overall accuracy when compared to the performance of SVM.

In summary, the SVM classifier’s margin-based optimization, coupled with its robust kernel methods and inherent noise resilience might have provided advantage over both single kNN and Ensemble (Subspace kNN) classifiers. This combination effectively harnessed the complex, nonlinear characteristics of fNIRS data, underscoring SVM’s suitability and reliability in emotion recognition tasks based on neurophysiological signals.

From an application standpoint, the consistently strong performance of SVM highlights a computationally efficient yet highly accurate approach to fNIRS-based emotion recognition. In scenarios demanding real-time classification—such as human–computer interaction or clinical neurofeedback—an SVM classifier can be deployed on modest hardware due to its relatively fast inference time once trained [10,29]. Furthermore, the high specificity across all algorithms reinforces the conclusion that the extracted time-domain features are indeed physiologically meaningful for emotional state differentiation. SVM’s particular advantage in handling nonlinear separability suggests that researchers might explore alternative kernel functions or hyperparameter tuning to further refine classification for more granular or overlapping emotional categories [17,44].

To sum up, our results indicate that SVM’s margin-focused, kernel-enabled approach is particularly well-suited to complex, high-dimensional fNIRS data obtained for emotion recognition. This method’s robustness, interpretability, and moderate computational cost underscore its feasibility for diverse real-time and clinical applications, pushing the envelope of single-modality brain–computer interfaces in detecting and classifying emotional states with both high accuracy and specificity.

### Limitations of the study and future directions

The sample size of obtained data was relatively small when compared to other application areas of machine learning (e.g., automation, finance). Nonetheless, previous emotion BCI studies conducted with fNIRS, and EEG modalities also trained their classification algorithms with similar sizes of training data. Future work will involve testing the proposed methodology on a larger subject cohort and higher number of labelled trials. We will also test the discriminative power of FNIRS-derived hemodynamic features obtained during a variety of naturalistic emotional states.

Deep learning (DL) approaches have shown promise for enhancing the accuracy of fNIRS-based BCI systems in classification, prediction or decoding of phenomenal states, when substantial amount of training data is available [52]. As a major strength, DL algorithms have the ability to capture the nuanced patterns in hemodynamic activity by use of advanced techniques applied for optimization of network architectures [53]. Indeed, previous work has successfully integrated DL models with both fNIRS and EEG data [54]. Since training DL models with training data sample size less than 5000 is reported to increase the risk of overfitting, our study did not incorporate DL algorithms because each emotional state was represented by a relatively small dataset [52]. Future studies will focus on evaluating DL methods for this classification problem using larger cohorts and data augmentation strategies to address the limitations posed by the small sample size of our study.

In clinical contexts, reliably detecting subtle shifts in emotional valence or arousal using fNIRS could support personalized mental health interventions, biofeedback for emotional regulation, or real-time monitoring in psychiatric settings [19,21]. For human–computer interaction, incorporating an SVM-based emotional state decoder can lead to more adaptive and responsive interfaces, enabling applications such as personalized learning environments or user-tailored entertainment systems [3,4]. As larger datasets become available, comparing SVM’s margin-based strength to the feature-learning capacity of deep neural networks would be an important next step. This could confirm whether SVM remains advantageous in data-limited contexts or whether neural architectures eventually surpass SVM once sufficient fNIRS data are amassed for stable training [10,52].

## Conclusion

The neurobiology of emotions is composed of complex coordinated interactions between the cerebral cortex and the subcortical regions. Despite advances in neuroscience research, the precise mechanisms by which human brain processes emotions are still not fully understood. Our approach leverages advanced machine learning techniques to achieve classification with over 90% accuracy, demonstrating that fNIRS alone is sufficient for effective emotional state detection in practical BCI applications. This unique contribution lies in our ability to use a lightweight and accessible technology, providing a scalable solution for real-world emotion recognition systems.

By leveraging computationally efficient supervised machine learning algorithms, specifically SVM, kNN, and Ensemble classifiers, we achieved significant accuracy in emotion decoding. Notably, our models achieved accuracies above 90% across all metrics in both binary (positive vs. negative) and multiclass (positive, neutral, negative) classifications. The comprehensive classification structure enhances the robustness of our model, supporting its applicability across varied emotional states.

Our findings highlight the robustness of fNIRS data in capturing physiologically informative signals that are less susceptible to subjective biases common in self-report methods. The high classification accuracies achieved without the need for deep learning techniques underscore the potential of classical machine learning algorithms in fNIRS-based emotion recognition.

This study advances the field of emotion recognition using fNIRS-based BCIs by providing a robust, single-session evaluation that captures significant effective differentiation with high accuracy. The use of non-invasive, cost-effective fNIRS technology combined with efficient machine learning classifiers presents a practical approach for real-world applications in mental health assessment, human–computer interaction, and adaptive technologies. Future research should aim to expand the participant pool to include diverse populations, test the system in real-world settings, and explore the integration of multimodal data sources, such as combining fNIRS with EEG. Additionally, investigating personalized models that account for individual differences in emotional processing could further enhance classification performance.

In conclusion, our study demonstrates that fNIRS, when coupled with classical machine learning algorithms, can effectively decode emotional states with high accuracy. This approach offers a practical and accessible solution for emotion recognition applications, circumventing the complexities associated with deep learning models. Our work contributes to the growing body of evidence supporting the viability of fNIRS as a standalone tool for emotion detection and underscores the effectiveness of computationally efficient algorithms in this domain.

## Supporting information

S1 PLOS One data. (XLSX)
